# Smart Bandwidth Assignation in an Underlay Cellular Network for Internet of Vehicles

**DOI:** 10.3390/s17102217

**Published:** 2017-09-27

**Authors:** Idoia de la Iglesia, Unai Hernandez-Jayo, Eneko Osaba, Roberto Carballedo

**Affiliations:** 1 DeustoTech-Fundacion Deusto, Deusto Foundation, Av. Universidades, 24, 48007 Bilbao, Spain; unai.hernandez@deusto.es (U.H.-J.); e.osaba@deusto.es (E.O.); roberto.carballedo@deusto.es (R.C.); 2 Facultad Ingeniería, Universidad de Deusto, Avda. Universidades, 24, 48007 Bilbao, Spain

**Keywords:** Internet of Vehicles, VANETs, smart wireless technologies, intelligent transportation systems, smart bandwith utilization, QoS requirements

## Abstract

The evolution of the IoT (Internet of Things) paradigm applied to new scenarios as VANETs (Vehicular Ad Hoc Networks) has gained momentum in recent years. Both academia and industry have triggered advanced studies in the IoV (Internet of Vehicles), which is understood as an ecosystem where different types of users (vehicles, elements of the infrastructure, pedestrians) are connected. How to efficiently share the available radio resources among the different types of eligible users is one of the important issues to be addressed. This paper briefly analyzes various concepts presented hitherto in the literature and it proposes an enhanced algorithm for ensuring a robust co-existence of the aforementioned system users. Therefore, this paper introduces an underlay RRM (Radio Resource Management) methodology which is capable of (1) improving cellular spectral efficiency while making a minimal impact on cellular communications and (2) ensuring the different QoS (Quality of Service) requirements of ITS (Intelligent Transportation Systems) applications. Simulation results, where we compare the proposed algorithm to the other two RRM, show the promising spectral efficiency performance of the proposed RRM methodology.

## 1. Introduction

Cooperative driving is expected to provide advance services in order to increase road safety, improve traffic management and create different new business opportunities for mobility services. In this way, the automobile industry is expected to increase their profit margin of Euro 54 billion in 2012 to Euro 79 billion by 2020 [1].

In the scenario of ITS, cooperative vehicular systems are a key factor not only to report information to the drivers in real time but also to gather accurate information about the traffic flow and events that occur during driving. Then, vehicles in a VANET can be considered as moving nodes that can address FCD (Floating Car Data), thanks to the creation of V2V and Vehicle-to-Infrastructure (V2I or I2V) links, giving rise to -ITS (Cooperative-ITS) [2]. In this context, the evolution of the Internet of Things (IoT) paradigm into new application scenarios as VANETs, introduced the definition of new terms such as Internet of Vehicles (IoV) [3] , creating a development framework that requires specific QoS attributes, as very a low latency to provide fast response to the events on the road [4].

Then, moving the concept of IoT into IoV , we find an ecosystem where vehicles (cars, trucks, motorbikes) and elements of the infrastructure (signals, sensors, actuators, etc.), besides pedestrians in an urban scenario, are interconnected thanks to an IP-based communication platform. Therefore, all these nodes (static and in movement at very different relative speeds) can generate, aggregate, consume and exchange information directly (using device-to-device communications) or indirectly (through a traffic management center, for example), with the goal of resolving different kinds of events, to result in providing a more safe, efficient and eco-friendly Intelligent Transport Systems.

As a result of this information exchange, vehicles can benefit from the information which might have been generated not only by surrounding information sources (sensors located on the road, elements of the infrastructure) but even by other vehicles that are located at a certain distance, for example, to obtain information about the traffic density on the road.

Nevertheless, a cooperative vehicular scenario like this must face multiple communication challenges, such as the adverse propagation conditions, high vehicles’ mobility pattern and their unpredictable direction of motion, not to mention the limited communication resources. Then, the technologies adopted to provide the communication architecture are a key factor that determine the performance of the network. Two candidate technologies are competing to enable V2X (Vehicle-to-Everything) communications: IEEE 802.11p and cellular network communications. Although IEEE 802.11p was specifically designed for V2X communications and to cater for ITS applications, it suffers from certain technical limitations. The main identified deficiency is poor performance in high vehicle density scenarios resulting from the MAC (Medium Access Control) layer implementation based on a regular IEEE 802.11 CSMA (Carrier Sense Multiple Access). Moreover, it does not guarantee the QoS which is essential to fulfill the different performance requirements of ITS applications [5,6].

In the scope of cellular networks, LTE-A supports D2D (Device-to-Device) functionality since 3GPP Rel.12, so it has already been tested in other IoT scenarios where nodes are static with the aim of maximizing the energy efficiency of the nodes. However, this technology was not meant to meet stringent V2X requirements in terms of latency, reliability and mobility. These limitations have motivated the research community to investigate cellular-based V2X communications. Additionally, D2D systems offer four different types of gain, i.e., proximity gain, hop gain, pairing gain and reuse gain. Based on these advantages, the applicability of D2D techniques for V2V communications has been analyzed in different works, such as [7]. In particular, there has been a reuse of gain stems from the fact that D2D and cellular links can share the same radio resources. Thus, in a D2D underlay cellular infrastructure, the same resources can be simultaneously utilized by the V-UE (Vehicular User Equipment) involved in V2V communication and by the ordinary C-UE (Common User Equipment), that is, a regular mobile user. This could improve the utilization of cellular spectrum and reduce the energy consumption of V2V communications. However, the reuse gain comes at the expense of increased interference level what in turn has to be neatly controlled by the network. In this context, the design of an efficient RRM for V2V communications which underlays the cellular network with minimal impact on cellular communications is a key problem. Therefore, efficient RRM is indisputably an element of paramount importance for deploying ITS applications with different QoS requirements.

Consequently, the motivation of this paper is to design an underlay RRM capable of improving cellular spectral efficiency while trying not to affect the performance of C-UEs’ communications. In this way, one of the contribution of this paper is that we design a three-step methodology to, first, mitigate the interferences generated by sharing radio resources between C-UEs and V-UEs and, secondly, to ensure the QoS requirements for the transmission of CAMs (Cooperative Awareness Messages) by vehicles. In this methodology, we, first, calculate which are the possible C-UE-V-UE pairs that could share radio resources; then, we define the transmission powers that satisfy both transmitters QoS requirements; and finally, using a metaheuristic, which gives an additional originality to our study, we select the pairs that allow us to provide a more efficient radio resource assignment than the RRMs we are comparing them against.

In addition, the main innovation of this paper is that, before applying the presented RRM, we organize the vehicles in clusters to reduce infrastructure connections and improve radio efficiency. That is, we understand each cluster as a moving zone to whom we assign variable-size pools depending on the transmission needs of vehicles of each cluster. In this way, the CH is the vehicle in charge of asking for resources to the infrastructure and distributing them among the CMs (Cluster Members). Therefore, the novelty of our proposal arises from how we allocate resources among V2X clusters with a presence of non-V2X UE (User Equipment)on the same bandwidth.

In the remaining sections, the paper is organized as follows. In Section 2, a literature review about RRM for D2D and V2V communications is presented. Section 3 presents the system model and problem definition. Next, Section 4 explains the Radio Resource Allocation procedure and Section 5 the simulation conditions and results obtained to validate our proposal. Finally, Section 6 presents a discussion about results and Section 7 presents the paper’s conclusions.

## 2. Literature

D2D communications have been proposed to allow nearby users to communicate with each other without passing through the infrastructure. It has enabled the devices to transmit at higher data rates, with lower power consumptions, lower latencies and improved spectral efficiency. Based on these advantages, the applicability of D2D techniques for V2V communications has been analyzed in different works, such as [7,8]. Khelil et al. [8] evaluated the suitability of existing D2D techniques for V2V communications and concluded that, in order to meet the stringent QoS of ITS applications, a careful adaption of the legacy D2D concepts is necessary to exploit them in the road safety domain.

A similar understanding prevails among the 3GPP community. As a consequence, in December 2015 a WI (Work Item) was approved [9] whose primarily goal was to standardize V2V system operating on the basis of LTE sidelink. D2D, specified in 3GPP Release 12 and 13, has been chosen to serve as a core technology but numerous areas for enhancement have been identified with the aim to fulfill V2V requirements. The most straightforward aspects that distinguish V2V from the pure D2D are related to the V-UEs’ velocity, the density of V-UEs and to the latency constraints. For example, one of the objectives of the V2V WI was to develop a solution capable of supporting relative velocities of up to 500 kph what inevitably implied changes to the physical layer structure. To cater for such rigorous requirements, reference signals’ density in time has been doubled in comparison to the legacy D2D. Other issues addressed in the course of this WI include, e.g., partitioning the relevant area into the zones with associated resource pools, targeted at minimizing the interference level and mitigating the “near-far” effect, or standardizing SPS (Semi-Persistent Scheduling) for sidelink transmissions in order to effectively serve a periodical part of V2V traffic. According to the official time-line, the WI is on the verge of completion (i.e., approval at September’s 3GPP RAN Plenary meeting), at least with respect to the feature subset concerning UE autonomous resource selection (so-called “Mode 2”).

Inside the extensive research carried out in the context of traditional D2D systems, e.g., [10,11,12] to refer to a few relevant papers, the work presented in [13] offers an excellent introduction to the underlay RRM problem. In this paper, authors propose an RRM solution which performs admission control and power allocation for each admissible D2D pair and its potential C-UE partners. Once they get all the admissible pairs and their corresponding power levels, they apply a maximum weight bipartite matching to define the final pairs.

Focusing our attention on V2X communications, Cheng et al. [7] study the coexistence of V2I and V2V communications in a D2D underlay mode and conclude that D2D in ITS exhibit transmission rate advantage with respect to the traditional V2V-only mode, the V2I-only mode, or the V2V overlay mode. Nevertheless, only a few studies have applied this underlay mode possibility to vehicular environments [7,8,14,15,16,17].

Botsov et al. [14] solve the interference problem originating from the reuse gain by dividing the cell coverage area into zones and assigning a pool of resources to each zone. With such approach, they guarantee a maximum acceptable interference level caused by C-UE in the V2V underlay. However, it suffers from a lack of scalability, it does not consider QoS requirements and its performance depends entirely on the zone definition and associated pool assignment. Xing et al. [15], instead of dividing the coverage area into zones and assigning pools, group together the road vehicles into multiple clusters. In their approach, each uplink resource can be allocated to only one cluster, that is, the number of resources that are reused by vehicles is in fact equal to the amount of clusters. As a result, they accomplish their primary aim which was to minimize the cellular radio resources consumption, but their approach does not satisfy QoS requirements for ITS applications deployment.

In traditional D2D systems, the performance objectives have typically been to maximize the overall network throughput of the existing C-UEs and admissible D2D pairs and to prioritize cellular links [11,18]. However, in vehicular environments most of the messages are relatively small and they have very strict requirements in terms of latency and reliability. Thus, the aforementioned performance will not be fulfilled for the deployment of ITS applications and, additionally, a number of other performance objectives have to be defined [16,17]. Wanlu et al. [17] propose an analytical method to transform latency and reliability requirements of V2V safety applications into optimization constraints. However, this concept does not consider the QoS requirements of other ITS applications. Ren et al. [16] study two different power control problems: (1) SPC (Sum Rate Oriented Power Control) aimed at maximizing the sum rate of all V2V links associated with infotainment services and (2) MPC (Minimum Rate Oriented Power Control) attempting to maximize the minimum achievable rate among all V2V links corresponding to the active safety applications which usually rely on small, fixed-size packets. Although in this approach the authors assume vehicles will spontaneously form clusters while traveling along the highway, they do not leverage the opportunities offered by clustering as they do not tie them with the resource reuse mechanism.

Once the performance objectives are defined, methodologies have to work out the RBAP (Resource Block Assignation Problem). Throughout history, it could be said that the most used method for solving the RBAP is the HA (Hungarian Algorithm) [19]. The HA is a combinatorial optimization algorithm originally designed for addressing assignment problems in a polynomial time. In this sense, it is interesting to point out that the HA is a deterministic algorithm. This method has been applied to RBAP problems in works such as [13,17] or [20]. Furthermore, HA has been applied in other fields such as transport [21] or economics [22]. In addition to the HA, some other alternatives have been proposed in the literature for solving similar problems as the RBAP. In [23], for example, two different methods are presented, the first one based on dynamic programming, and the second one consisting on a greedy algorithm. On the other hand, in [23], a distributed dynamic spectrum protocol is developed.

Although, as can be seen in the literature, HA is the most used algorithm for solving RBAP, this method presents some drawbacks. First of all, HA is an exact approach with a high complexity for large scenarios. Additionally, as it has been mentioned before, HA is a deterministic method. This is not a disadvantage by itself, but as it is demonstrated below, the presence of some randomness leads to higher quality solutions. There are many algorithms in the scientific community which base their execution on the application of some kind of randomness. Arguably, heuristics and metaheuristics [24] are the most used and well-known methods that can be classified in this group. Concretely, we have decided to use a metaheuristic for the resolution of the RBAP. A metaheuristic is an optimization technique that solves a specific problem using only general information and knowledge common to a wide variety of optimization problems with similar characteristics. Furthermore, metaheuristics explore the solution space in order to achieve good optimization results with independence of the problem. In this sense, the main reason of the utilization of a metaheuristic is its adaptability, and its easy implementation. Additionally, metaheuristics are flexible techniques, and their efficiency has been proved in a wide range of fields [25].

To summarize this section, we propose a more comprehensive methodology for underlaying RRM than the works presented hitherto in the literature. Accordingly, we organize vehicles in clusters because instead of assigning fixed radio resource pools to static zones as in [14] do, variable-size pools are assigned to moving vehicle clusters. Therefore, as we explained at the end of the Introduction section, the novelty of our proposal emerges from how we allocate radio resources among V2X clusters with a presence of non-V2X UE on the same bandwidth. In this way, in our proposal the CH is in charge of asking for resources for all the vehicles of the cluster and, then, distribute the resource polls among them. Finally, and to the best of our knowledge, it is interesting to point out that this is the first time in the literature that the presented kind of metaheuristic is utilized for solving this RBAP, which gives an additional originality to our study.

## 3. Problem Definition

In this section, we first introduce the system model and, then, the resource allocation problem for V2V communication inside vehicular clusters is formulated.

### 3.1. System Model

The scope of this work is focused on how radio resources can be reused for V2V communications underlying cellular network, where in *M* V2V communications coexist with *N* cellular C-UE communications. In this system, C-UEs and V-UEs share the available uplink resources and only V-UEs utilize D2D communications.

Through our research, we propose to share uplink resources as the interference generated on the cellular links affects only to the eNodeB-side which is assumed to possess advanced signal processing capabilities to mitigate such detrimental phenomena. Furthermore, the interference suffered by the V2X links can be handled through spatial separation. On the other hand, if the downlink reuse was implemented, the transmission from the eNodeB could have serious negative impacts on the V-UE receivers, because it could violate the reliability requirement of safety applications, which are the main focus of this paper. Additionally, most of the existing studies consider the UL (uplink) resources to support D2D communications since there are some regulatory restrictions in certain regions for reusing the DL (downlink) resources [26].

**Figure 1:**
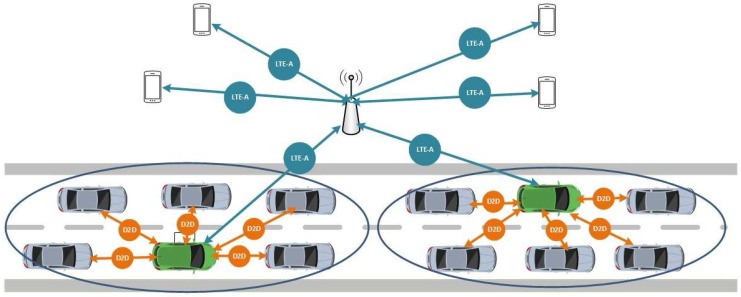
Overview of the system.

The proposed system, shown in Figure 1, can be decomposed into three main components: (i) users; (ii) scenario and (iii) network systems, whose properties vary over a number of dimensions. There are two different type of users: (1) C-UEs which are the typical cellular users and (2) V-UEs which are the vehicles. These users move in the given scenario and have access to network infrastructure resources, called RBs (Resource Blocks). They use these RBs to send their messages to other users but, depending on the type of user and the type of message, they send them though the network infrastructure or directly, by means of V2V communication link.

One feature of our proposal is that V-UEs are organized into clusters. Considering that clustering is a technique for grouping devices in the geographical vicinity to make the network more robust and scalable, it is very common in VANETs to find vehicles organized in clusters for deploying ITS applications. These clusters are usually defined based on some common characteristics such as vehicle movement, velocity, direction or transmission range, among others [27].

In our proposed method, as the clustering creation is not the aim of our research, we define a simple centralized clustering algorithm based on the vehicle with the highest number of neighbor vehicles, meaning a neighboring vehicle in its coverage area. The steps to define the CHs, and then, the CMs, are represented by means of Algorithm 1. Therefore, using the information about the V-UEs’s position which is known by a central server (called GeoServer), our algorithm organizes V-UEs into clusters. Each cluster is formed by one CH, which is the most famous vehicle, and some CMs, its neighbors.

**Algorithm 1:** Clustering algorithm. 
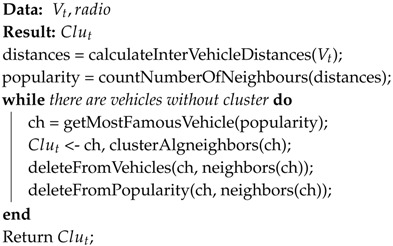


At this point, each cluster is formed by one CH and some CMs. The CH is responsible of updating the infrastructure with relevant information of the cluster and updating its CMs with relevant information received from the infrastructure. In this way, the novelty of this clustering strategy comes from the fact that in this case, instead of each V-UE requests for resources to the eNodeB, the CH (Cluster Head) is the user in charge of requesting RB for all its CMs’ transmissions.

Therefore, let $RB_{t}:=\left\{rb_{1},rb_{2},\ldots ,rb_{k}\right\}$ be the set of uplink orthogonal RBs that could be assigned in one frame. This $RB_{t}$ set of orthogonal RBs is divided into different resource pools, $RP_{t}:=\left\{rp_{1},rp_{2},\ldots ,rp_{k}\right\}$ , where each $rp_{x}$ is formed by a variable number of RBs depending on the existing transmission requirements.

Formally, the set of C-UEs, V-UEs and clusters in the scenario at time interval *t* can be define as $C_{t}:=\left\{c_{1},c_{2},\ldots ,c_{n}\right\}$, $V_{t}:=\left\{v_{1},v_{2},\ldots ,v_{m}\right\}$ and $CLU_{t}:=\left\{clu_{1},clu_{2},\ldots ,clu_{j}\right\}$, respectively. During the observation or simulation period, additional users may join or leave the scenario, subject to their routes, which may vary with V-UEs’ and C-UE’ densities over time and space. In addition, the properties of the scenario impact users’ mobility and communications’ reliability.

Furthermore, the network system comprises the cellular infrastructure and GeoServer which manages the creation and maintenance of clusters and where some ITS applications are implemented, e.g., congestion prediction, planning of routes, etc.

### 3.2. Problem Formulation

This paper addresses an efficient radio resource assignment in an underlay cellular network where V-UEs communications coexist with cellular C-UEs communications. We denote this assignment efficient because we aim for improving the spectral efficiency of the underlay network by maximizing the number of users served with the minimum number of radio resources.

In the same way as in [16], we study two different resource sharing situations: (1) SPC when V-UE asks for an infotainment transmission and (2) MPC for safety, management and efficiency ITS applications transmissions. However, in this paper we will focus only on transmission on CAM transmissions, which follow the second situation. We decided to center this research in CAMs because information distributed by CAM Management is commonly used by related use cases, e.g., Approaching Emergency Vehicle or Slow Vehicle Warning, and therefore the CAM Management is a mandatory facility [28].

Therefore, we will have a multi-objective optimization problem with two different terms: one related with C-UE optimization conditions and the other one associated to CAM transmissions QoS requirements. The first term is defined by the maximization of C-UE’s sum-rate Equation (1), which is a common performance requirement.

(1) $$Th^{C-UE}=\sum_{n=1}^{N}\log_{2}\left(1+\varepsilon_{n}^{c}\right)$$

being $\varepsilon_{n}^{c}$ the SINR (Signal-to-Interference-plus-Noise Ratio) of the C-UE-eNodeB link.

On the other hand, CAM’s QoS requirements can be defined as a MPC problem, so the other term of the optimization problem is defined by Equation (2)

(2) $$Th^{D2D}=min_{k\epsilon \{1,\ldots ,M\}}\log_{2}\left(1+\varepsilon_{m}^{d}\right)$$

## 4. Methodology

In this section, we introduce the RRM methodology proposed in this paper to improve the spectral efficiency of users’ communications. The main problem it has to resolve is to optimize the spectral efficiency being capable of mitigating the interferences and ensuring the different QoS requirements of ITS applications.

**Figure 2:**
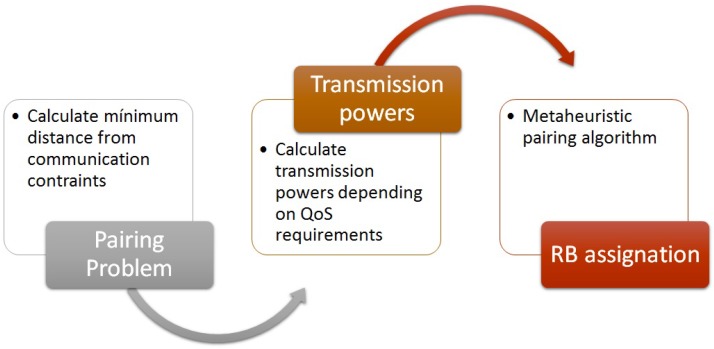
Resource Allocation steps.

Therefore, with the intention of solving the overall problem, we divide the main one into three subproblems, which are shown in Figure 2.

1. Pairing Problem: The first one is the pairing problem, in which in order to determine if a C-UE and a V-UE can share the same RB to transmit their messages, we calculate the minimum distance there should be between a C-UE and a V-UE transmitter to mitigate the interference. Knowing this distance, the eNodeB can select which are all the possible pairs.
2. Transmission Power Allocation: After solving the pairing problem, we continue with the second subproblem which is the transmission power assignation for each possible pair of C-UE and V-UE transmissions. Thereby, we have a bipartite graph with all the possible pairs with their respective transmission powers. With this transmission powers, it is easy to calculate the achievable throughput of the pair of communications which will be considered the weight of each pair in the bipartite graph.
3. RB Assignation: Having reached this point, we move to the third subproblem, where we end up with the RB assignation. To solve this combinatorial optimization subproblem we have design a specific parallel meta-heuristic.This subproblems are explained in the following subsections.

### 4.1. Minimum Distance between C-UE and V-UE Transmitter

The eNodeB can only assign the same RB to a C-UE and a V-UE if constraints Equations (3)–(6) are satisfied. Let $\varepsilon_{n}^{c}$ and $\varepsilon_{m}^{d}$ denote the SINR of C-UE-eNodeB *n* communication and V2V *m* communication, respectively, $P_{n}^{c}$ and $P_{m}^{d}$ denote the transmit power of the C-UE *n* and V-UE transmitter *m*, respectively, and $g_{nB}$, $g_{m}$, $h_{mB}$, $h_{mn}$ denote channel gains of the links that participate in the system, being *n* the C-UE, *m* the V-UE transmitter and *B* the eNodeB.

(3) $$\varepsilon_{n}^{c}=\frac{P_{n}^{c}*g_{nB}}{\sigma^{2}+P_{m}^{d}*h_{mB}}\geq \varepsilon_{min}^{c}$$

(4) $$\varepsilon_{m}^{d}=\frac{P_{m}^{d}*g_{m}}{\sigma^{2}+P_{n}^{c}*h_{mn}}\geq \varepsilon_{min}^{d}$$

(5) $$P_{n}^{c}\leq P_{max}^{c}$$

(6) $$P_{m}^{d}\leq P_{max}^{d}$$

These constraints mean that the minimum SINR requirement have to be guaranteed for both communications Equations (3) and (4) and the power transmission of the C-UE and V-UE must be lower than the maximum permitted Equations (5) and (6). The power of additive white Gaussian noise on each channel is assumed to be $\sigma^{2}$.

Moreover, there are also minimum transmission powers that the C-UE Equation (7) and the V-UE Equation (8) have to fulfill in order to guarantee the message achieves the receiver. The transmission power is minimum when there is no interference and we only guarantee the minimum SINR.

(7) $$P_{n,min}^{c}=\frac{\varepsilon_{n,min}^{c}*\sigma^{2}}{g_{nB}}$$

(8) $$P_{m,min}^{d}=\frac{\varepsilon_{m,min}^{d}*\sigma^{2}}{g_{m}}$$

Therefore, the values of transmission power the system has to assign to C-UE Equation (9) and V-UE Equation (10) are limited by the minimum transmission power which depends on the propagation losses and the maximum transmission power which depends on the LTE-Advanced configuration parameters.

(9) $$P_{n,min}^{c}<P_{n}^{c}\leq P_{max}^{c}$$

(10) $$P_{m,min}^{d}<P_{m}^{d}\leq P_{max}^{d}$$

**Figure 3:**
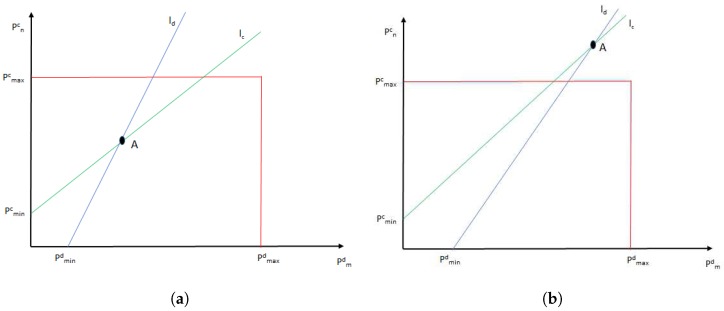
Representation of pairing constraints. (**a**) possible pair; (**b**) impossible pair.

The pairing process can be easily understood in Figure 3 where the rectangle delimits maximum transmission powers for C-UE and V-UE and lines $l_{c}$ and $l_{d}$ represents constraints Equations (3) and (4).

The area on the right of $l_{d}$ are the possible combinations of transmission powers that fulfill restriction Equation (4) and the area over $l_{c}$ are the possible combinations of transmission powers that meet Equation (3). Therefore, the area between both lines contains all the possible power transmission combinations which fulfill all the constrains needed for sharing a RB between C-UE and a V-UE. That is, if the intersection of both lines, point A, is inside the rectangle of maximum transmission powers, C-UE and V-UE can be paired, as it is shown in Figure 3a. However, if the intersection is outside the rectangle, as in Figure 3b, those C-UE and V2V pair cannot share the same RB fulfilling all the constraints.

Moreover, from Figure 3 it is deduced that point A comprises the pair of minimum transmission powers that fulfill all the pairing constraints. Since the equations of $l_{d}$ and $l_{c}$ are Equations (11) and (12), respectively,

(11) $$P_{m}^{d}=\frac{g_{m}}{\varepsilon_{min}^{d}*h_{nm}}*P_{m}^{d}-\frac{\sigma^{2}}{h_{nm}}$$

(12) $$P_{n}^{c}=\frac{\varepsilon_{min}^{c}*h_{mB}}{g_{nB}}*P_{m}^{d}+\frac{\varepsilon_{min}^{c}*\sigma^{2}}{g_{nB}}$$

calculating the intersection of both lines the coordinates of point A Equation (13) are obtained.

(13) $$\left\{\begin{array}{c} P_{A}^{c}=\frac{\sigma^{2}\left(g_{j}\varepsilon_{min}^{c}+\varepsilon_{min}^{c}\varepsilon_{min}^{d}h_{mB}\right)}{g_{j}g_{nB}-\varepsilon_{min}^{d}h_{mB}h_{nm}}\\ P_{A}^{d}=\frac{\sigma^{2}\left(g_{nB}\varepsilon_{min}^{d}+\varepsilon_{min}^{c}\varepsilon_{min}^{d}h_{nm}\right)}{g_{j}g_{nB}-\varepsilon_{min}^{d}h_{mB}h_{nm}} \end{array}\right.$$

Owing to fading affects randomly influencing the LTE-A signal quality with time, geographical position or radio frequency, we take into account this random property in the parametrization of the channel link. As we consider both the fast fading due to multi-path propagation and slow fading due to shadowing, the channel gain between C-UE *n* and the eNodeB *B* can be expressed as

(14) $$h_{nB}=K\cdot \beta_{nB}\cdot \zeta_{nB}\cdot d_{nB}^{-\alpha }$$

where *K* is a constant determined by system parameters, $\beta_{i,B}$ is fast fading gain with exponential distribution, $zeta_{i,B}$ is the slow fading gain with log-normal distribution, α is the path-loss exponent, and $d_{\left(i,B\right)}$ is the distance between C-UE *n* and the eNodeB. In the same way, we can define the channel gain of V2V communication as $g_{m}$, the channel gain between the transmitter *m* of the V2V pair and the eNodeB as $h_{mB}$, and the channel gain between C-UE *n* and V-UE transmitter *m* as $h_{mn}$.

Thus, having the minimum transmission powers for C-UE and V-UE, the minimum distance between C-UE and V-UE transmitter that can satisfy all the constraints can be calculated getting the value of $h_{nm}$ from $P_{A}^{c}$ and $P_{A}^{d}$, and next, finding the values of $d_{nm}$. Knowing the minimum distance it is easier for the eNodeB to pairing C-UEs and V-UE.

$$L_{nm}=\left\{\begin{array}{cc} {\left(\frac{k\beta_{nm}\zeta_{nm}\varepsilon_{min}^{d}\varepsilon_{min}^{c}P_{max}^{c}h_{mB}}{P_{max}^{c}g_{nB}g_{m}-\sigma^{2}\varepsilon_{min}^{c}\left[g_{m}-\varepsilon_{min}^{d}*h_{mB}\right]}\right)}^{\frac{1}{\alpha }} & \mathrm{if}\frac{P_{max}^{c}*g_{nB}}{\sigma^{2}+P_{max}^{d}*h_{mB}}\geq \varepsilon_{min}^{c}\\ {\left(\frac{k\beta_{nm}\zeta_{nm}\varepsilon_{min}^{d}\varepsilon_{min}^{c}\left[P_{max}^{c}h_{mB}+\sigma^{2}\right]}{g_{nB}\left[P_{max}^{d}g_{m}-\varepsilon_{min}^{d}\sigma^{2}\right]}\right)}^{\frac{1}{\alpha }} & \mathrm{if}\frac{P_{max}^{c}*g_{nB}}{\sigma^{2}+P_{max}^{d}*h_{mB}}\geq \varepsilon_{min}^{c} \end{array}\right.$$

### 4.2. Transmission Power Allocation

In the previous subsection we have addressed the possible pairing of C-UE and V-UE. Once we have finished the pairing process and obtained the possible pairs, we have to assign the transmission power for each user using the formula defined by Equation (15)

(15) $$P_{tx}=\min\left(P_{RBmax},\frac{P_{o}}{PathGain*\alpha }\right)$$

where $P_{RBmax}$ is the maximum transmission power each C-UE or V-UE is allowed to use per RB, $P_{o}$ is the minimum threshold for RSRP (Reference Signal Received Power), PathGain is attenuation of the signal as it propagates through space from source to destination, and α is the pathloss compensation factor that can vary from 0.1 to 1.

### 4.3. RB Allocation: XueBlockSolver Algorithm

In this section, the algorithm developed to face the designed radio resource assignation problem RBAP is described. In this case, we have formulated the RBAP as a combinatorial optimization problem [29]. Combinatorial optimization is one of the most studied fields in artificial intelligence, optimization, logistics, and other applications. Multiple research works are published annually in this area, both in journals [30], and conferences [31], and also in books [32]. Different sort of problems exist within this kind of optimization. In this specific study, we have modeled the RBAP as a combinatorial design problem [33]. In line with this, many different metaheuristics can be found in the literature that are able to deal with this type of problems. In this sense, some of these methods are based on a single search, such as Simulated Annealing [34] and Tabu Search [35], and some others are based on a multiple search (population based algorithms), such as genetic algorithm [36], and the ant colony optimization [37]. Metaheuristics can also be classified in search based algorithms and constructive algorithms. Search based algorithms start from an initial complete solution or an initial set of complete solutions which are modified until reaching a final solution, while constructive algorithms start from a partial solution or a set of partial solutions which are built until reaching an final complete solution.

In this study, a population based search algorithm has been designed for solving the RBAP. The philosophy of the presented technique is to have a population of autonomous individuals, which perform single local searches in parallel until the ending criterion is reached. For this reason, the developed technique has been called Multiple and Parallel Block Solver Algorithm (MP-BSA).

As it has been mentioned, the RBAP is formulated as a combinatorial design problem. The objective of this problem is to find a configuration which guarantees the QoS, minimizing the number of resource blocks used and maximizing the total throughput. Thus, the problem has been treated as a hierarchical multi-objective one, with a two-leveled objective function. In order to understand this objective function, it is convenient to clarify that we count with three different sets, containing the throughput of every singles CUE and VUE, and every CUE-VUE pair:

$$X:\{T\_CUE_{1},T\_CUE_{2},T\_CUE_{3},\;\ldots ,T\_CUE_{n}\}$$

$$Y:\{T\_VUE_{1},T\_VUE_{2},T\_VUE_{3},\;\ldots ,T\_VUE_{m}\}$$

$$Z:\{T\_VUE\_CUE_{1},T\_VUE\_CUE_{2},\;\ldots ,T\_VUE\_CUE_{l}\}$$

Now, the two-leveled objective function that we should minimize is as follows:

(16) $$\sum_{i=1}^{n}x_{i}\;\sum_{j=1}^{m}y_{j}\;\sum_{k=1}^{l}z_{k}$$

(17) $$\sum_{i=1}^{n}T\_CUE_{i}x_{i}\;\sum_{j=1}^{m}T\_CUE_{j}y_{j}\;\sum_{k=1}^{l}T\_VUE\_CUE_{k}z_{k}$$

The first objective (Equation (16)) is to minimize the number of resource block used. After that, and considering solutions with the same number of resource blocks, the objective is to maximize the total throughput (Equation (17)). To correctly understand these functions, it should be pointed out that $x_{i}$, $y_{i}$ and $z_{i}$ are binary variables, which are 1 if the corresponding CUE, VUE and VUE-CUE pair are present in the solution.

As can be read in some studies of the literature [38], something important when any algorithm is developed, or operator, is the codification used to represent the partial, or complete solutions of the problem. For this reason, the codification chosen has to be clearly described, since depending on the representation used, some operators can be developed or not. In this study, the permutation representation has been utilized. Thus, each solution is represented by a permutation of the different elements of the environment. Additionally, each resource block is divided by a semicolon, and the elements of the same pair by a coma. As an example, and taking into account a system with four CUEs and four VUEs, one possible solution could be represented in the following way:

X:{CUE_02,VUE_03;CUE_01,VUE_04;

CUE_03,VUE_01;CUE_04;VUE_02}

In this case, this specific solution is composed by three pairs ({CUE_02,VUE_03}, {CUE_01,VUE_04}, {CUE_03,VUE_01}) and two lone elements (CUE_04 and VUE_02).

The working way of the developed MP-BSA has been summarized in the Algorithm 2. As can be seen in this algorithm, the first steps of the MP-BSA is to randomly generate the initial population and to define the above described objective function. After that, an iterative process starts, in which every individual of the population performs a small modification on its structure. If this newly generated solution improves the previous one, it is accepted. In any other case, the new solutions is discarded. For the modification of individuals, the well-known exchange operator has been used, which has been widely used in other fields [39]. For this movement, first, two elements of the solution are randomly selected, in this case the elements in boldface. Then, the position of these two elements are exchanged. After this interchange, the blocks are built again. This step is used with the aim of no generating infeasible solutions. For this reason, it may be that new solutions have a different number of blocks than the previous ones. Taking the previous individual *X*, and assuming, for example, CUE_01 and CUE_04 have been randomly selected:

X:{CUE_02,VUE_03;CUE_01,VUE_04;

CUE_03,VUE_01;CUE_04;VUE_02}

One possible new individual could be the following one:

$$X^{\prime }:\{CUE\_02,VUE\_03;\mathrm{CUE}\_04,VUE\_04;$$

CUE_03,VUE_01;CUE_01;VUE_02}

Finally, this iterative process is repeated until the termination criteria is reached. In this case, this termination criteria is composed by two conditions: a fixed number of total generations and a number of generations with no improvements in the best found solution. If any of these conditions are fulfilled, the algorithm finishes its execution and returns it best individual.

**Algorithm 2:** Pseudocode of the proposed MP-BSA. 
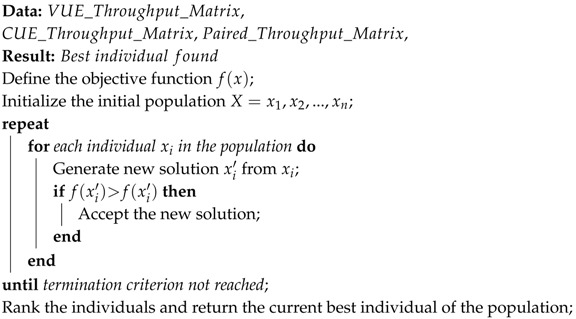


## 5. Proposed Solution Tests and Validation

In this section, we present the scenarios and metrics used to validate our underlay RRM proposal presented in previous sections and provide system-level simulation results for evaluating its performance.

### 5.1. Scenarios and Parameters

We assume a single cell outdoor system with a carrier frequency of 2 GHz where each RB has a bandwidth of 180 KHz for the uplink communication. Radio resources are organized in 100 RBs following the SC-FDMA (Single Carrier Frequency Divison Multiple Access) scheme used in the UL [40]. According to the agreed 3GPP V2X deployment scenario for highways [41], a straight highway with 6 lanes in total, 3 in each direction, passes through the cell, and *M* V-UEs and *N* C-UEs are distributed in the scenario defined to check the proposed solution. The number of V-UEs and C-UEs varies depending on the scenarios simulated explained in Section 5.2 and V-UEs move with a speed of 140 km/h. The used channel models are specified by [42] and the simulation parameters are summarized in Table 1.

### 5.2. Performance Metrics and Baseline Methods

Proposed solution will be evaluated using three metrics, which will provide a set of results that allow us to analyze the performance of the solution:

- Throughput, defined as the average number of bits per second transmitted through users’ links. In other words, the sum of the data rates that are delivered by all users considered in the tested scenarios.
- Spectral Efficiency, defined as the average number of bytes transmitted per RB. Spectral Efficiency is a key metric as the radio spectrum is a limited resource. As it was introduced in the problem definition section, the main aim of our proposal is to define an underlay RRM capable of increasing the spectral efficiency of the cellular networks for giving access to a higher number of users with the same resources.
- Energy Efficiency, defined as the average number of bytes transmitted per Watt consumed. This metric evaluates how the RRM methodology takes benefit from the transmission power used.

Previous metrics are evaluated from two different points of view:

1. Results obtained from V2V communication links to analyze the communication performance offered to V-UEs by the RRM methodology.
2. Results obtained from all the communication links to evaluate the communication performance offered to all the users of the scenario by the proposed RRM methodology.

As it has been exposed in Section 1, the design of an efficient underlaying RRM for co-existing C-UEs and V-UEs users with minimal impact on cellular communications is a key problem. Therefore, we evaluate the previous metrics depending on C-UEs’ type of traffic, interpreted as the variation of transmitted C-UEs’ packet size. This variable allows to analyze how the V-UEs’ links performance depends on C-UEs’ traffic pattern.

To carry out the analysis, the scenarios presented in Table 2 have been defined, where it has been taken into consideration the relationship between the number of C-UEs and V-UEs in the scenario, with the total number of users being constant and equal to 50 users. This analysis enables us to evaluate in which situations the proposed RRM method performs better, in the ones where predominate the number of C-UEs or, on the contrary, where the number of V-UEs is predominant.

To obtain a realistic analysis, we compare our RRM proposal with the following already tested and checked methods:

1. Underlay RRM methodology which maximizes the sum rate of both C-UE and V-UE using the possibility of sharing both users the same RB for the transmission.
2. Overlay RRM methodology which maximize the sum rate of users using one RB for each transmission with the maximum transmission power.

### 5.3. Simulation Results

This section introduces and analyzes the results obtained according to C-UEs’ traffic pattern. These outputs are discussed at Section 6. Therefore, 15 groups of simulation instances have been carried out by varying in each group the size of packets transmitted by C-UEs. Each group of instances have been evaluated in the nine scenarios defined in Table 2.

**Figure 4:**
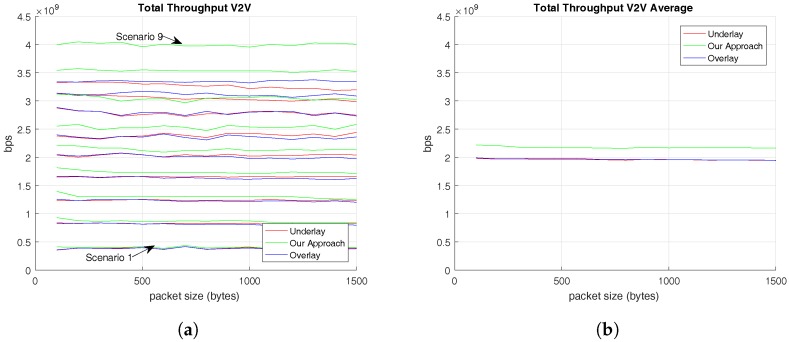
Throughput offered to V-UEs based on C-UEs’ traffic pattern. (**a**) Average Throughput of V-UEs in different scenarios; (**b**) Total Average Throughput of V-UEs.

The analysis starts with the presentation of throughput results. First of all, Figure 4 shows the throughput offered to V-UE users by the three different RRM algorithms evaluated. On the one hand, Figure 4a shows the average throughput reached by V-UEs in each scenario. On the other hand, Figure 4b exposes the total average throughput given to V-UEs by each RRM algorithm according to C-UEs’ traffic pattern. Figure 4 shows that the RRM proposed in this paper provides higher throughput values to V-UE users than the other RRM algorithms that have been evaluated.

In addition, Figure 4 shows us that values of V-UEs’ throughput are independent of the C-UEs’ traffic pattern. This conclusion comes from the linear trend followed by the V-UEs’ throughput results of all the RRM solutions evaluated. The reason is that V-UE packets always follow the same traffic pattern defined by 3GPP TR 36.885 v14.0.0 (2016-06), which defines the CAM traffic pattern followed by V-UEs. This traffic pattern consists of periodically transmitting first a packet of 300 bytes followed by 4 of 190 bytes.

In addition, by delving into results of Figure 4a, it is noticed that while the number of V-UEs increases, the throughput given to V-UEs rises, since the maximum throughput value is achieved at Scenario 9, where there are 45 V-UEs and 5 C-UEs, and the minimum value is accomplished at Scenario 1, where 5 V-UEs and 45 C-UEs coexist.

Table 3 summarizes the average V-UE’ throughput results showed in Figure 4b. These values demonstrate the previous conclusions because, first of all, values show that the three RRM solutions are linear according to C-UEs’ packet size and, secondly, they evidence that the highest throughput average value of V-UEs is achieved by the RRM solution proposed in this paper.

**Figure 5:**
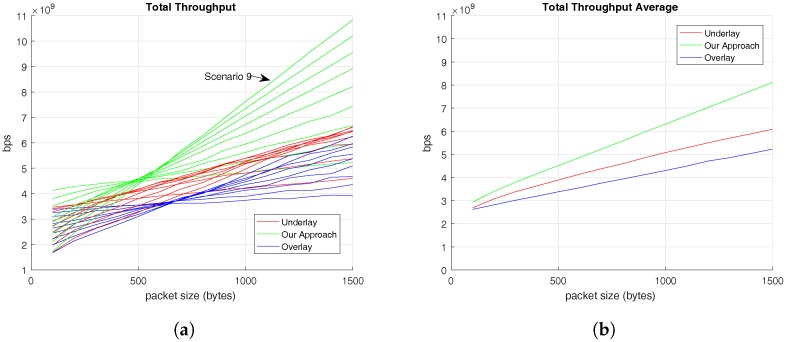
Throughput offered to V-UEs based on C-UEs’ traffic pattern. (**a**) Total Average Throughput in different scenarios; (**b**) Total Average Throughput for all set of users.

Figure 5 presents the total average throughput offered to the set of users , C-UEs and V-UEs, according to the C-UEs’ packet size in the nine previously explained scenarios. Values of total throughput for all the set of users in each scenario is presented in Figure 5a. Nevertheless, Figure 5b shows the total average values obtained in each RRM solution for all users according to the C-UEs’ packet size.

Figure 5a shows that the three evaluated RRM solutions follow a similar and increasing trend which coincides with the increment of the C-UEs packet size. This increasing trend is understandable because the bigger the C-UE packet is, the higher number of resources it requests. This trend is also clear in Figure 5b, which additionally shows that the RRM algorithm proposed in this paper provides higher values of throughput to the users. In addition, looking into results from Figure 5a, we obtain that the highest throughput accomplished is in the Scenario 9, where 5 V-UEs and 45 C-UEs coexist. This is owing to C-UEs generating a higher volume of information than V-UEs. Finally, Figure 5b reveals that comparing the other RRM evaluated alternatives, the underlay solution offers higher throughput than the overlay one. These conclusions are revealed again in Table 4. In the manner of Table 3, Table 4 presents the average throughput offered to all the users by the different RRM solutions analyzed.

**Figure 6:**
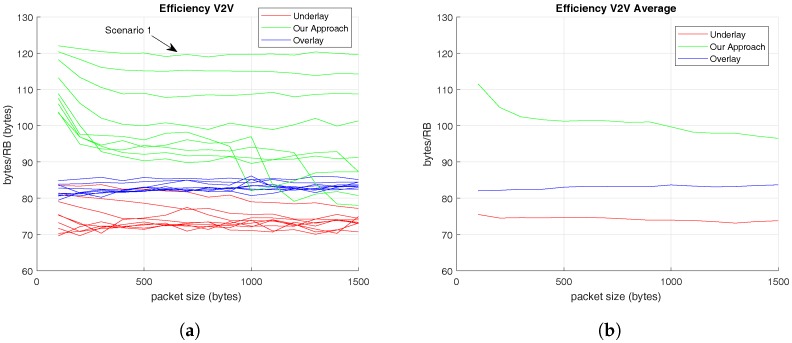
Spectral Efficiency offered to V-UEs based on C-UEs’ traffic pattern. (**a**) Total Average Spectral Efficiency for V-UEs in different scenarios; (**b**) Total Average Spectral Efficiency for V-UEs.

Figure 6 displays the spectral efficiency achieved by V2V links. In this case, Figure 6a shows the average spectral efficiency reached by V2V links in each evaluated scenario and Figure 6b presents the total average spectral efficiency of these V2V communications taking into account the results of all the scenarios.

Figure 6a shows that the maximum value of spectral efficiency delivered by the proposed RRM solution is achieved at the Scenario 1, where the number of V-UEs is the highest one (45) and the number of C-UEs is the lowest one (5). This proves that the proposed methodology performs better when the number of V-UEs is high, which demonstrate this solution is focused on increasing the spectral efficiency of V2V links. In addition, Figure 6b reveals that the average spectral efficiency offered by the RRM methodology proposed in this paper is higher than the methodologies which it is compared to. Table 5 sums up these average values of V2V spectral efficiency represented in Figure 6b, and shows that the total average spectral efficiency of V2V communications reaches its highest value with the presented RRM. In addition, Table 5 reveals that the maximum value of spectral efficiency for V2V links is accomplished when the C-UEs’ message size is 100 bytes using the RRM presented in this paper. In other words, while the C-UEs’s packet size increases, the spectral efficiency of V2V links decreases. However, this does not happen in the overlay RRM solution since it assigns individual RBs to each transmission which makes it independent of the rest of the users’ transmissions.

**Figure 7:**
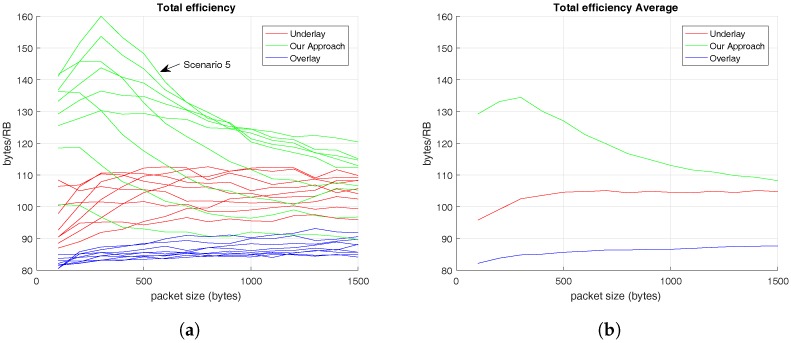
Spectral Efficiency offered to V-UEs based on C-UEs’ traffic pattern. (**a**) Total Average Spectral Efficiency in different scenarios; (**b**) Total Average Spectral Efficiency for all set of users.

To end up with the spectral efficiency analysis, Figure 7 presents the total spectral efficiency of the solutions evaluated. In this way, Figure 7a shows the total spectral efficiency results obtained in the different scenarios and, Figure 7b reveals the total average results for the set of users.

Figure 7a shows that the proposed RRM reaches its maximum spectral efficiency value at Scenario 5, where 25 C-UEs and 25 V-UEs coexist. This fact evidences that our aim is to minimize the number of RBs used, or in other words, maximize the number of pairs which is easier when there is the same number of C-UEs and V-UEs.

Figure 7b provides evidence that our proposal offers the highest spectral efficiency compared with the other alternatives evaluated. Therefore, according to the results obtained, the presented RRM methodology is able to take advantage of RBs sending a higher number of bytes than the alternatives. This fact allows us to provide a service to a higher number of users, or at least, to provide better service to the users. Table 6 summarizes the results presented in Figure 7b and remarks the better performance of the RRM solution proposed in this paper. As it is defined in Table 1, the maximum spectral efficiency per RB is 180 bytes. Therefore, from Figure 7a where the maximum value reached by our RRM proposal is 160 bytes, we could say this proposal accomplishes the 88.89% of the maximum spectral efficiency value and, on average, it reaches 66.32%.

**Figure 8:**
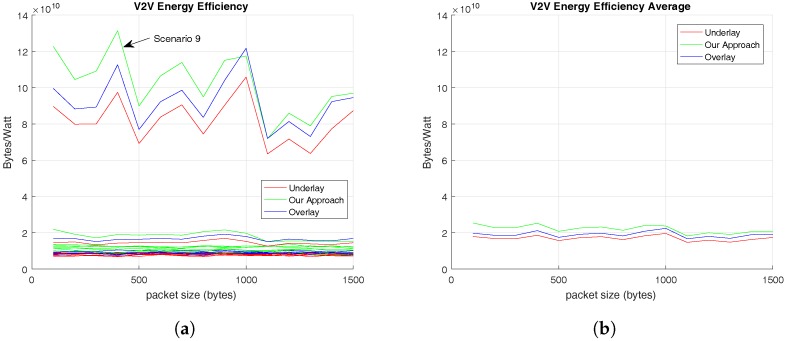
Energy Efficiency offered to V-UEs based on C-UEs’ traffic pattern. (**a**) Total Average Energy Efficiency for V-UEs in different scenarios; (**b**) Total Average Energy Efficiency for V-UEs.

This section finishes with the analysis of energy efficiency, i.e., the number of bytes transmitted per watt. First, Figure 8 shows the energy efficiency offered to V2V links. On the one hand, Figure 8a presents the energy efficiency of V-UEs’ transmissions in each scenario and, on the other hand, Figure 8b reveals the total average results of this metric for the different RRM evaluated.

In Figure 8a, similar results are presented for all the scenarios except at the Scenario 9, which provides higher energy efficiency. Furthermore, Figure 8b demonstrates that the proposed solution provides higher energy efficiency for V2V links than the other RRM methodologies evaluated. These values are summarized in Table 7 which also evidences that the energy efficiency of V2V communications is not related with the C-UEs’ packet size.

**Figure 9:**
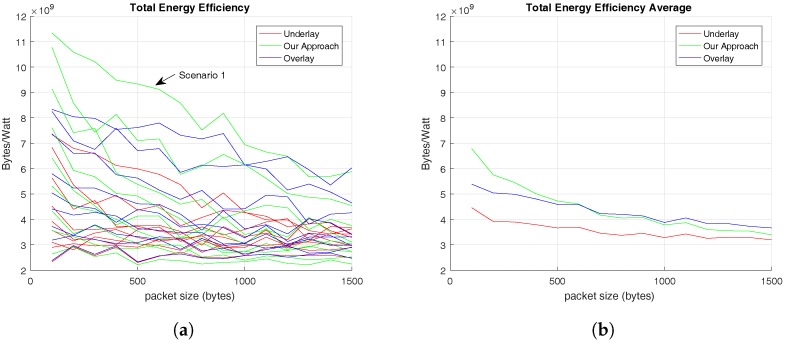
Energy Efficiency offered to V-UEs based on C-UEs’ traffic pattern. (**a**) Total Average Energy Efficiency in different scenarios; (**b**) Total Average Energy Efficiency for all set of users.

Finally, Figure 9 presents the total energy efficiency offered by the RRMs to users. In this way, Figure 9a shows the total energy efficiency in each scenario and Figure 9b presents the total average energy efficiency for all the scenarios.

In Figure 9a, the highest values for energy efficiency are reached in Scenario 1, where there are 5 C-UEs and 45 V-UEs. This means that the energy efficiency of V2V communications is higher than the common cellular communications, which makes sense because the distance between transmitter and receiver in a V2V communication is usually lower than a cellular one. However, Figure 9b shows that in some cases the overlay RRM offers higher energy efficiency than the RRM proposed in this paper. This is owing to the fact that overlay RRM does not have to mitigate interferences between cellular and V2V users. This values are exposed in Table 8 and provide evidence that for small C-UEs’ packet size, from 100 to 600 bytes, the energy efficiency offered by our RRM proposal performs better than the overlay one, but, for bigger C-UEs’ packet size, the overlay RRM offers better performance. In any case, for the simulations carried out in this evaluation the total average energy efficiency offered by our RRM proposal is higher that the overlay one.

## 6. Discussion

The aim of this discussion section is to summarize the meaningful results presented in the previous section. Regarding the throughput results, we could have validated that V-UEs’ throughput is independent of the C-UEs’ traffic pattern. This conclusion comes from the linear trend followed by the V-UEs’ throughput for all the different C-UEs’ packet size, as it is shown in Figure 4. In addition, the total throughput results provide evidence that the RRM we propose provides the highest values of total throughput, as it is presented in Figure 5.

On the other hand, in Figure 6, the V2V’s efficiency results prove that the proposed methodology performs better when the number of V-UEs is high and that V2V’s efficiency is also independent of C-UEs’s traffic pattern. Additionally, the total efficiency results demonstrate that our approach is more efficient than the other alternatives, as it is demonstrated in Figure 7.

The last metric evaluated is the energy efficiency, which results shown in Figure 8 provide evidence that the proposed methodology performs better than the compared ones. In addition, the energy efficiency of V2V links is independent of the C-UEs’ traffic pattern. On the other hand, the total energy efficiency results provide evidence that energy efficiency of V2V communications is higher than the common cellular ones, which is a coherent consequence of a V2V communication because the distance between transmitter and receiver is usually lower than in a cellular one.

Finally, Table 9 collects the average results obtained for the presented metrics.

## 7. Conclusions

The possibility of using D2D underlay cellular technology for VANETs could improve the utilization of cellular spectrum and reduce the energy consumption of V2V (Vehicle-to-Vehicle) communications. Although the reuse gain comes at the expense of increasing the interference level, it is possible to be neatly controlled by the network designing an RRM to mitigate the interferences and ensure the different QoS requirements of ITS applications.

In this paper, firstly, we briefly analyze various concepts presented hitherto in the literature related to efficient sharing of the available radio resources between common cellular and V2X communications. Secondly, this paper presents the design of an underlay RRM methodology capable of improving cellular spectral efficiency while having minimal impact on cellular communications. Finally, we provide system-simulations results, which evidence the improvement of spectral efficiency provided by the presented methodology. As future work, in order to improve the energy efficiency results obtained, we plan to design a more accurate power transmission assignment, which will reduce the interference generated.

## Figures and Tables

**Table 1:** Simulation Parameters.

Parameter	Value
Carrier Frequency	2 GHz
Number of RBs	100
RB bandwidth	180 kHz
Scenario	1 km highway
Number of users	50
V-UEs velocity	140 kmph
Channel model	Line of Sight
V2V coverage radio	100 m
Number of Scenarios	9
Simulation time	2 s
Number of runs per scenario	6
Frame size	1 ms
$P_{RBmax}$	125 mW
$P_{o}$	−162.5 dBm
α	0.8

**Table 2:** Definition of Scenarios.

Scenario	C-UEs	V-UEs
1	5	45
2	10	40
3	15	35
4	20	30
5	25	25
6	30	20
7	35	15
8	40	10
9	45	5

**Table 3:** Average Throughput offered to V-UEs based on C-UEs’ traffic pattern.

CUE’s Message Size (Bytes)	Underlay V2V Throughput (Gbps)	Our Approach V2V Throughput (Gbps)	Overlay V2V Throughput (Gbps)
100	1.9798	2.2196	1.9911
200	1.9681	2.2081	1.9744
300	1.9684	2.1794	1.9757
400	1.9676	2.1745	1.9801
500	1.9650	2.1674	1.9820
600	1.9635	2.1700	1.9746
700	1.9556	2.1634	1.9600
800	1.9498	2.1617	1.9623
900	1.9636	2.1783	1.9613
1000	1.9586	2.1671	1.9548
1100	1.9558	2.1698	1.9579
1200	1.9491	2.1673	1.9521
1300	1.9542	2.1688	1.9526
1400	1.9489	2.1670	1.9546
1500	1.9474	2.1652	1.9456
Media	1.9597	2.1752	1.9653

**Table 4:** Average Total Throughput achieved based on C-UEs’ traffic pattern.

CUE’s Message Size (Bytes)	Underlay Total Throughput (Gbps)	Our Approach Total Throughput (Gbps)	Overlay Total Throughput (Gbps)
100	2.6870	2.9351	2.6186
200	3.0729	3.3967	2.8166
300	3.3820	3.7943	3.0143
400	3.6363	4.1614	3.1914
500	3.8890	4.5104	3.3809
600	4.1432	4.8688	3.5563
700	4.3750	5.2224	3.7543
800	4.5923	5.5784	3.9307
900	4.8477	5.9575	4.1163
1000	5.0782	6.3053	4.3020
1100	5.2871	6.6725	4.4930
1200	5.5013	7.0332	4.7124
1300	5.7040	7.3952	4.8591
1400	5.8899	7.7527	5.0409
1500	6.0869	8.1100	5.2257
Media	4.5448	5.5796	3.9342

**Table 5:** Average Spectral Efficiency offered to V-UEs based on C-UEs’ traffic pattern.

CUE’s Message Size (Bytes)	Underlay V2V Spectral Efficiency (Bytes)	Our Approach V2V Spectral Efficiency (Bytes)	Overlay V2V Spectral Efficiency (Bytes)
100	75.5158	111.5009	82.0601
200	74.5242	105.0593	82.1765
300	74.6291	102.4324	82.4734
400	74.5778	101.6482	82.4722
500	74.6811	101.2451	83.0456
600	74.6977	101.4136	83.2602
700	74.5434	101.3483	83.2362
800	74.2527	100.9259	83.1750
900	73.9347	101.0398	83.1834
1000	73.9485	99.6729	83.6675
1100	73.8607	98.2533	83.3569
1200	73.5458	97.9194	83.1306
1300	73.1310	97.9186	83.2180
1400	73.5731	97.1496	83.5048
1500	73.7803	96.5138	83.6841
Media	74.2130	100.9361	83.0430

**Table 6:** Average Total Spectral Efficiency (bytes) achieved based on C-UEs’ traffic pattern.

CUE’s Message Size (Bytes)	Underlay V2V Spectral Efficiency (Bytes)	Our Approach V2V Spectral Efficiency (Bytes)	Overlay V2V Spectral Efficiency (Bytes)
100	95.7937	129.2273	82.1711
200	99.1588	133.1300	83.8041
300	102.5021	134.4520	84.8239
400	103.5603	130.0844	85.0603
500	104.5878	127.0249	85.6566
600	104.7855	122.7010	85.9722
700	105.0782	119.7708	86.3758
800	104.3845	116.7125	86.3510
900	104.8364	114.8894	86.6161
1000	104.5595	113.0139	86.5990
1100	104.4074	111.5848	86.8759
1200	104.9000	110.9248	87.2326
1300	104.4592	109.7956	87.4499
1400	105.0987	109.2632	87.5854
1500	104.7921	108.1939	87.6160
Media	103.5269	119.3846	86.0127

**Table 7:** Average Energy Efficiency offered to V-UEs based on C-UEs’ traffic pattern.

CUE’s Message Size (Bytes)	Underlay V2V Energy Efficiency (Bytes)	Our Approach V2V Energy Efficiency (Bytes)	Overlay V2V Energy Efficiency (Bytes)
100	17.905	25.398	19.747
200	16.839	22.829	18.611
300	16.755	22.859	18.645
400	18.610	25.262	21.175
500	15.699	20.797	17.476
600	17.307	22.655	19.176
700	17.837	23.179	19.706
800	16.221	21.363	18.259
900	18.305	23.995	20.832
1000	19.625	23.778	22.438
1100	14.714	18.259	16.685
1200	15.911	19.996	18.002
1300	14.817	19.092	16.931
1400	16.303	20.620	18.934
1500	17.384	20.643	19.225
Media	16.949	22.048	19.056

**Table 8:** Average Total Energy Efficiency achieved based on C-UEs’ traffic pattern.

CUE’s Message Size (Bytes)	Underlay V2V Energy Efficiency (Bytes)	Our Approach V2V Energy Efficiency (Bytes)	Overlay V2V Energy Efficiency (Bytes)
100	4.4618	6.7902	5.3890
200	3.9224	5.7585	5.0450
300	3.8978	5.4545	4.9905
400	3.7932	5.0135	4.8062
500	3.6656	4.7267	4.5924
600	3.6905	4.6072	4.5828
700	3.4600	4.1699	4.2278
800	3.3731	4.0570	4.2021
900	3.4523	4.0740	4.1365
1000	3.2847	3.7835	3.8812
1100	3.4272	3.8718	4.0592
1200	3.2558	3.6018	3.8385
1300	3.2977	3.5524	3.8314
1400	3.2870	3.5393	3.7257
1500	3.1950	3.3954	3.6664
Media	3.5643	4.4264	4.3317

**Table 9:** Summary of Average Simulation Results.

Metrics	Underlay	Our Approach	Overlay
**V2V Throughput (Gbps)**	1.9597	2.1752	1.9653
**Total Throughput (Gbps)**	4.5448	5.5796	3.9342
**V2V Spectral Efficiency (bytes)**	74.2130	100.9361	83.0430
**Total Spectral Efficiency (bytes)**	103.5269	119.3846	86.0127
**V2V Energy Efficiency (Gb/W)**	16.949	22.048	19.056
**Total Energy Efficiency (Gb/W)**	3.5643	4.4264	4.43317

## Acronyms List

**CAM**	Cooperative Awareness Message
**CH**	Cluster Head
**C-ITS**	Cooperative-ITS
**CM**	Cluster Member
**CSMA**	Carrier Sense Multiple Access
**C-UE**	Common User Equipment
**DL**	downlink
**D2D**	Device-to-Device
**ETSI**	European Telecommunications Standards Institute
**FCD**	Floating Car Data
**FDD**	Frequency Division Duplexing
**HA**	Hungarian Algorithm
**I2V**	Infrastructure-to-Vehicle
**IoT**	Internet of Things
**IoV**	Internet of Vehicles
**ITS**	Intelligent Transportation Systems
**LOS**	Line of Sight
**LTE-A**	LTE-Advanced
**LTE**	Long Term Evolution
**MAC**	Medium Access Control
**MPC**	Minimum Rate Oriented Power Control
**QoS**	Quality of Service
**RB**	Resource Block
**RBAP**	Resource Block Assignation Problem
**RRM**	Radio Resource Management
**RSRP**	Reference Signal Received Power
**SC-FDMA**	Single Carrier Frequency Divison Multiple Access
**SINR**	Signal-to-Interference-plus-Noise Ratio
**SPC**	Sum Rate Oriented Power Control
**SPS**	Semi-Persistent Scheduling
**UE**	User Equipment
**UL**	uplink
**V2I**	Vehicle-to-Infrastructure
**V2V**	Vehicle-to-Vehicle
**V2X**	Vehicle-to-Everything
**VANET**	Vehicular Ad Hoc Network
**V-UE**	Vehicular User Equipment
**VRU**	Vulnerable Road User
**WI**	Work Item
